# Evaluation of six blood-based age prediction models using DNA methylation analysis by pyrosequencing

**DOI:** 10.1038/s41598-019-45197-w

**Published:** 2019-06-20

**Authors:** Antoine Daunay, Laura G. Baudrin, Jean-François Deleuze, Alexandre How-Kit

**Affiliations:** 1 Laboratory for Genomics, Foundation Jean Dausset – CEPH, Paris, France; 2 Laboratory of Excellence GenMed, Paris, France; 3 Centre National de Recherche en Génomique Humaine, CEA, Institut François Jacob, Evry, France

**Keywords:** Methylation analysis, DNA methylation, DNA methylation

## Abstract

DNA methylation has been identified as the most promising molecular biomarker for the prediction of age. Several DNA methylation-based models have been proposed for age prediction based on blood samples, using mainly pyrosequencing. These methods present different performances for age prediction and have rarely, if ever, been evaluated and intercompared in an independent validation study. Here, for the first time, we evaluate and compare six blood-based age prediction models (Bekaert^1^, Park^2^, Thong^3^, Weidner^4^, and the Zbiec-Piekarska 1^5^ and Zbiec-Piekarska 2^6^), using DNA methylation analysis by pyrosequencing on 100 blood samples from French individuals aged between 19–65 years. For each model, we perform correlation analysis and evaluate age-prediction performance (mean absolute deviation (MAD) and standard error of the estimate (SEE)). The best age-prediction performances were found with the Bekaert and Thong models (MAD of 4.5–5.2, SEE of 6.8–7.2), followed by the Zbiec-Piekarska 1 model (MAD of 6.8 and SEE of 9.2), while the Park, Weidner and Zbiec-Piekarska 2 models presented lower performances (MAD of 7.2–8.7 and SEE of 9.2–10.3). Given these results, we recommend performing systematic, independent evaluation of all age prediction models on a same cohort to validate the different models and compare their performance.

## Introduction

Aging is a natural biological process present in most living organisms and characterized by the progressive decline of several molecular, cellular and physiological functions that are influenced by both genetic and environmental factors^1,2^. Several studies have aimed to identify potential biological and/or molecular biomarkers of aging that correlate with chronological age and could be used in prediction models to estimate the chronological age of individuals^3,4^. Such prediction models could be particularly useful in forensic science and for public health concerns^3,4^.

DNA-based age prediction models rely on four types of DNA biomarkers of aging: telomere length, mitochondria mutations, single joint T-cell receptor excision circle (sjTREC) rearrangements and DNA methylation. Telomere shortening has been shown to be associated with aging and the replicative senescence of the cells characterized by the Hayflick limit^5^, and the inverse correlation between telomere length and chronological age has been used for age prediction based on DNA extracted from blood or teeth^3,6,7^. Similarly, the accumulation of mutations in mitochondrial DNA (mtDNA), induced by oxidative stress damage, has also been associated with aging and metabolic senescence of the cells^8,9^, and a large deletion of mtDNA accumulated during the aging process has been used for age prediction but with poor performance^7,10^. More recently, sjTREC loss has also been associated with aging^11^ and has been used to predict chronological age based on blood samples, using real-time PCR^12–15^.

Epigenetic alterations, including histone modifications and DNA methylation, have also been suggested as a hallmark of aging^1^. Two types of changes in DNA methylation have been characterized during aging: (i) the epigenetic drift, which corresponds to the progressive divergence of the methylome between individuals that occurs with increasing age, and (ii) the epigenetic clock, which is based on modifications of the methylome that correlate to chronological age similarly in every individual and are used in age prediction models^16^. DNA methylation-based age-prediction models have been developed principally for use with blood samples and are based either on a low number of CpGs (DNA methylation biomarkers), using locus-specific technologies such as pyrosequencing, or on a higher number of CpGs requiring the use of genome-wide epigenotyping array technologies^17–19^. These DNA methylation models outperform both the previously described DNA-based age prediction models and the RNA and protein-based age prediction models, thus making DNA methylation the most promising molecular age-prediction biomarker^4,20^.

As a consequence, several pyrosequencing blood-based age-prediction models have been developed which use DNA methylation analysis by pyrosequencing, and present the advantage of requiring only a small number of analyzed CpGs, which is particularly useful for forensic applications. Some models were developed after initial screening for the best age-prediction biomarkers, using whole-genome epigenotyping array data^21,22^ due to the high correlation of DNA methylation quantification between epigenotyping arrays and pyrosequencing^23,24^, while other models were developed from a lower number of candidate genes using pyrosequencing analysis without genome-wide pre-screening^25–28^. Notably, while most of the models are based on at least 3 different loci, one model has been developed as a single locus model and uses only two GpGs located in a gene known as *ELOVL2*^27^. Notably, this gene has been identified as one of the best age prediction biomarkers and has thus been integrated into several age prediction models^21,25–28^. It should also be noted that the majority of the models predict age according to a multivariate linear equation, with the exception of one model which relies on a multivariate quadratic equation as it considers a quadratic relationship between *ELOVL2* DNA methylation and chronological age^25^. Other recurring DNA methylation-based, age prediction biomarkers used in different models include *ASPA*, *KLF14* and *TRIM59*^22,25,26,28^.

While these blood-based age prediction models, which use DNA methylation analysis by pyrosequencing, all present a good level of age-prediction accuracy (mean absolute deviation from chronological age (MAD) of around 3–5 years)^17^, to date few have been evaluated in other studies by other laboratories^26,29^, and no study has evaluated and compared different models in the same population. Here we present an evaluation of six blood-based age prediction models using DNA methylation analysis by pyrosequencing on 100 blood samples from 100 French individuals aged from 19 to 65 years. Taking these six models, namely the models of Bekaert^1^, Park^2^, Thong^3^, Weidner^4^, Zbiec-Piekarska 1^5^ and Zbiec-Piekarska 2^6^, we began by implementing all the pyrosequencing assays using DNA standards with known DNA methylation values and we evaluated the correlation between the DNA methylation value of each CpG and the chronological age obtained with our cohort. Next, we evaluated and compared the correlation between the chronological age and the predicted age obtained with the models, as well as the performance of each age prediction model measured by the mean absolute deviation (MAD), the standard error of the estimate (SEE) and the percentage of correct age prediction, using a threshold of 5, 7.5 and 10 years difference between the predicted and chronological age. The individuals were also grouped according to gender and assigned to one of three age groups: Young adults (19–35 years), Middle-aged adults (35–49 years) and Older adults (50–65 years), in order to evaluate the performance of the six chosen models on the basis of gender and of three age categories.

## Material and Methods

### Human blood samples

The study was conducted in accordance with current ethical and legal frameworks. Anonymized blood samples were obtained after informed consent from healthy donors through French blood bank, EFS (Etablissement Français du Sang, Paris, France – research agreement 15/EFS/012). All methods were performed in accordance to the recommendations of the French National Committee of Ethics (Comité Consultatif National d’Ethique pour les Sciences de la Vie et de la Santé). Peripheral blood samples were derived from 100 healthy French donors (42 women and 58 men) aged from 19–65 years. Individuals were assigned to one of three groups according to their chronological age: Group I comprising young adults (aged 19–34 years, n = 34), Group II comprising middle-aged adults (ages 35–49 years, n = 33), and Group III comprising older adults (ages 50–65 years, n = 33) (Supplementary Fig. 1). Buffy coats were obtained from blood after 10 min centrifugation at 1600g and frozen at −80 °C before DNA extraction.

### DNA extraction and quantification

DNA extraction was performed on buffy coats using the QIAmp DNA blood mini Kit (Qiagen) on a QIAcube robotic workstation (Qiagen) according to the manufacturer’s instructions. DNA quantification was performed using the Qubit^TM^ dsDNA HS assay Kit on a Qubit 3 Fluorometer (Thermo Fischer Scientific) according to the manufacturer’s instructions.

### Bisulfite conversion

Bisulfite conversion of DNA was performed on 1 µg of genomic DNA, using the EpiTect Bisulfite Kit 48 (Qiagen) on a QIAcube robotic workstation (Qiagen) according to the manufacturer’s instructions. Bisulfitetreated DNA was diluted to a final concentration of 20 ng/µl for DNA methylation analysis by pyrosequencing.

### PCR amplification

The PCR primer pairs and their annealing temperatures (Ta) used for PCR on bisulfite- treated DNA are given in Supplementary Table 1.

The regions of interest were amplified in 20 µL PCR reactions in a Mastercyler Pro S (Eppendorf) using 20 ng of bisulfite-treated DNA as a template. The PCR mix included 1× HotStar Taq DNA polymerase buffer, 1.8 mM of additional MgCl_2_, 200 µM of each dNTP, 200 nM of each primer (800 nM for *PDE4C*) and 2 U of HotStar Taq DNA polymerase. Cycling conditions included an initial denaturation step performed for 10 min at 95 °C, followed by 50 cycles of 30 sec denaturation at 95 °C, 30 sec annealing at Ta and 30 sec elongation at 72 °C. The final step included 5 min elongation at 72 °C.

### DNA methylation analysis by pyrosequencing

The pyrosequencing primers and corresponding sequences for analysis by pyrosequencing are given in Supplementary Table 1.

10 µl of PCR product was purified and prepared for pyrosequencing according to a previously described protocol^30,31^. DNA methylation analysis was performed using PyroMark Gold SQA Q96 Kit (Qiagen) on a PyroMark Q96 MD (Qiagen) and analyzed with PyroMark CpG software (Qiagen). DNA methylation data of the 100 blood samples from French individuals are available in Supplementary Dataset.xlsx.

### Statistical analysis and graphical representation

All statistical analysis and graphical representations were performed using R (https://www.r-project.org/) and MS Excel (Microsoft). The correlation between chronological age and DNA methylation predicted age was assessed using the Pearson r correlation coefficient. The coefficient of determination R² was calculated as the square of the r coefficient. For each age prediction model, the mean absolute deviation (MAD), the standard error of the estimate (SEE) and the percentage of correct predictions were calculated.

## Results

### Implementation of the eleven pyrosequencing assays and the six blood-based age prediction models

**Figure 1:**
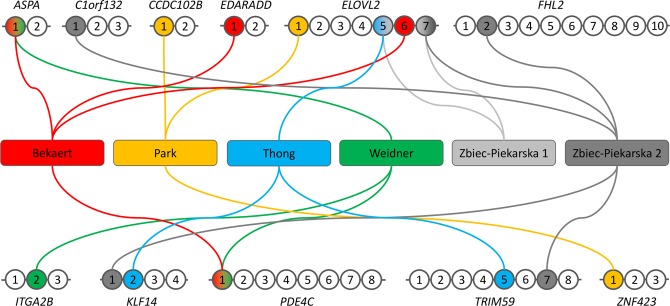
Description of the CpGs included in the six blood-based age prediction models using DNA methylation analysis by pyrosequencing.

Six blood-based age prediction models using pyrosequencing for DNA methylation analysis were selected for evaluation on blood samples from 100 French donors aged between 19 and 65 years. The selected models were those of Bekaert^1^, Park^2^, Thong^3^, Zbiec-Piekarska 1^5^, Zbiec-Piekarska 2^6^, and Weidner4, which use 4, 3, 3, 2, 5 and 3 CpG sites in 4 (*ASPA*, *EDARADD, ELOLV2* and *PDE4C*), 3 (*CCDC102B*, *ELOVL2* and *ZNF423*), 3 (*ELOVL2*, *KLF14* and *TRIM59*), 1 (*ELOVL2*), 5 (*C1orf132*, *ELOVL2*, *FHL2*, *KLF14* and *TRIM59*) and 3 (*ASPA*, *ITGA2B* and *PDE4C*) genes of interest respectively (Fig. 1). In total, 11 genes including 52 CpG sites were analyzed by pyrosequencing (Supplementary Tables 1 and 2). Contrary to the original studies, the DNA extractions were performed on buffy coats instead of whole blood. However this modification should not impact the DNA methylation analysis, as the buffy coat is the main source of DNA in whole blood.

The 11 different, previously published, pyrosequencing assays were first evaluated against standards of known degrees of DNA methylation (0, 25, 50, 75 and 100%) in order to determine their efficiency and linearity, and to detect any possible PCR-induced biases (Supplementary Figs 2 and 3). All pyrosequencing assays presented an observed DNA methylation of the 0% and 100% DNA methylation standards close to the expected values, with a higher variability of the observed value of the 100% standard for some CpG sites (Supplementary Figs 2 and 3). The 25%, 50% and 75% DNA methylation standards presented a quantification close to or slightly lower than their expected value, with the notable exception of the *PDEC* assay for which all the observed values were close to 0 (Supplementary Figs 2 and 3). In most assays, these results indicated the presence of only a slight PCR bias in favor of the unmethylated allele, however this bias was very strong for the *PDE4C* gene. To also evaluate the possible amplification biases induced by the use of different PCR cycles, we performed replicate experiments for the eleven assays with the same bisulfite-treated commercial DNA sample using either 45 or 50 PCR cycles (Supplementary Fig. 3). The DNA methylation values obtained for all CpGs included in the age-prediction models were very similar for both experimental conditions and presented no statistically significant differences (Supplementary Fig. 3), indicating that the use of 45 or 50 cycles of PCR should not modify the quantification of DNA methylation or the prediction of age.

The correlation analysis of DNA methylation of all CpGs and the chronological age of all individuals revealed a strong correlation present overall (mean absolute r = 0.640), which was stronger for the CpGs included in the six age-prediction models (mean absolute r = 0.758), although these CpGs correlations were not systematically the strongest within a given region (Supplementary Fig. 5 and Table 1). It should also be noted that all the CpGs of *ITGA2B* presented a weak correlation (−0.464≥ r ≥−0.325 while all the CpGs of *ELOVL2*, 4 of which are included in 5 different age prediction models, presented a very strong correlation (0.742≥ r ≥0.862); this explains 55.1% to 74.3% of the age variance in our group of individuals (Supplementary Fig. 5 and Table 1). The Pearson correlation coefficient of the CpGs included in the six age prediction models was very similar between men and women, with the exception of *ASPA* and *C1orf132* which presented a difference of 0.165 and 0.166 respectively in their r coefficients (Table 1).

**Table 1:** Correlation between chronological age and DNA methylation for all CpGs analyzed.

Gene Symbol	CpG	All		Men		Women	
		r	R²	r	R²	r	R²
*ASPA*	1	**−0.635**	**0.403**	**−0.681**	**0.464**	−0.516	0.267
	2	−0.591	0.349	−0.601	0.361	**−0.550**	**0.302**
*C1orf132*	1	−0.677	0.458	−0.743	0.552	−0.577	0.333
	2	**−0.700**	**0.490**	−0.726	0.528	**−0.643**	**0.414**
	3	−0.699	0.489	**−0.754**	**0.568**	−0.597	0.356
*CCDC102B*	1	**−0.672**	**0.452**	**−0.682**	**0.465**	**−0.686**	**0.470**
	2	−0.573	0.328	−0.537	0.289	−0.638	0.408
*EDARADD*	1	**−0.747**	**0.558**	**−0.770**	**0.593**	**−0.713**	**0.508**
	2	−0.675	0.456	−0.677	0.458	−0.658	0.433
*ELOVL2*	1	0.742	0.551	0.755	0.570	0.710	0.504
	2	0.790	0.624	0.799	0.638	0.763	0.582
	3	0.833	0.694	0.829	0.688	0.830	0.689
	4	0.831	0.691	0.818	0.670	0.847	0.717
	5	0.785	0.616	0.769	0.592	0.805	0.648
	6	**0.862**	**0.743**	**0.852**	**0.726**	**0.877**	**0.769**
	7	0.794	0.630	0.782	0.611	0.807	0.652
*FHL2*	1	**0.795**	**0.632**	**0.775**	**0.601**	0.822	0.676
	2	0.753	0.567	0.724	0.525	0.791	0.625
	3	0.764	0.583	0.722	0.521	0.837	0.700
	4	0.782	0.612	0.747	0.558	**0.838**	**0.702**
	5	0.742	0.550	0.712	0.507	0.780	0.608
	6	0.698	0.488	0.652	0.425	0.770	0.593
	7	0.626	0.392	0.592	0.350	0.674	0.454
	8	0.672	0.452	0.652	0.425	0.691	0.478
	9	0.660	0.436	0.596	0.356	0.761	0.579
	10	0.561	0.315	0.514	0.264	0.634	0.402
*ITGA2B*	1	**−0.464**	**0.215**	**−0.465**	**0.216**	**−0.421**	**0.177**
	2	−0.341	0.116	−0.359	0.129	−0.278	0.077
	3	−0.325	0.106	−0.315	0.099	−0.319	0.102
*KLF14*	1	**0.768**	**0.590**	**0.765**	**0.586**	**0.789**	**0.622**
	2	0.693	0.480	0.700	0.491	0.666	0.444
	3	0.618	0.382	0.657	0.432	0.509	0.259
	4	0.499	0.249	0.480	0.231	0.514	0.265
*PDE4C*	1	**0.757**	**0.574**	**0.772**	**0.597**	**0.721**	**0.519**
	2	0.459	0.210	0.473	0.224	0.392	0.154
	3	0.468	0.219	0.518	0.269	0.321	0.103
	4	0.296	0.087	0.156	0.024	0.495	0.245
	5	0.524	0.275	0.566	0.320	0.467	0.218
	6	0.377	0.142	0.342	0.117	0.415	0.172
	7	0.429	0.184	0.419	0.175	0.450	0.203
	8	0.361	0.130	0.402	0.161	0.245	0.060
*TRIM59*	1	0.570	0.325	0.592	0.350	0.505	0.255
	2	0.570	0.325	0.543	0.295	0.631	0.398
	3	0.730	0.533	0.695	0.483	0.788	0.622
	4	0.695	0.482	0.694	0.481	0.680	0.462
	5	**0.758**	**0.575**	**0.759**	**0.576**	0.748	0.560
	6	0.755	0.570	0.729	0.532	**0.790**	**0.624**
	7	0.739	0.547	0.719	0.516	0.774	0.599
	8	0.652	0.425	0.638	0.407	0.672	0.452
*ZNF423*	1	**−0.648**	**0.420**	**−0.670**	**0.449**	**−0.584**	**0.342**
	2	−0.551	0.303	−0.625	0.390	−0.427	0.183
	3	−0.586	0.344	−0.621	0.385	−0.495	0.245

For each category and each gene, the strongest correlation is indicated in **bold**. The CpGs included in one of the six age prediction models are underlined.

The formulas used to predict age in the six different age prediction models given in Table 2 were obtained from previous studies^21,22,28^ or personal communications by the authors of the models (Bekaert, Thong and Zbiec-Piekarska 1 age prediction models).

**Table 2:** Formulas of the different age prediction models used.

Age prediction model	Formula
Bekaert	26.444119 − 0.201902 × *ASPA* (CpG_1_) − 0.239205 × *EDARADD* (CpG_1_) + 0.0063745 × *ELOVL2* (CpG_6_)² + 0.6352654 × *PDE4C* (CpG_1_)
Park	39.73167 − 0.69994 × *CCDC102B* (CpG_1_) + 1.19242 × *ELOVL2* (CpG_1_) − 0.28914 × *ZNF423* (CpG_1_)
Thong	−20.372 + 0.830 × *ELOVL2* (CpG_5_) + 1.723 × *KLF14* (CpG_2_) + 0.715 × *TRIM59* (CpG_5_)
Weidner	38.0 − 0.264 × *ASPA* (CpG_1_) − 0.237 × *ITGA2B* + 1.647 (CpG_2_) × *PDE4C* (CpG_1_)
Zbiec-Piekarska 1	−42.8393176902677 + 0.63266203860581 × *ELOVL2* (CpG_5_) + 0.877474742612866 × *ELOVL2* (CpG_7_)
Zbiec-Piekarska 2	3.26847784751817 − 0.355450171437202 × *C1orf132* (CpG_1_) + 0.465445549010653 × *ELOVL2* (CpG_7_) + 0.237081243617191 × *FHL2* (CpG_2_) + 0.832684435238792 × *KLF14* (CpG_1_) + 0.306488541137007 × *TRIM59* (CpG_7_)

### Evaluation and comparison of the six blood-based age prediction models

**Figure 2:**
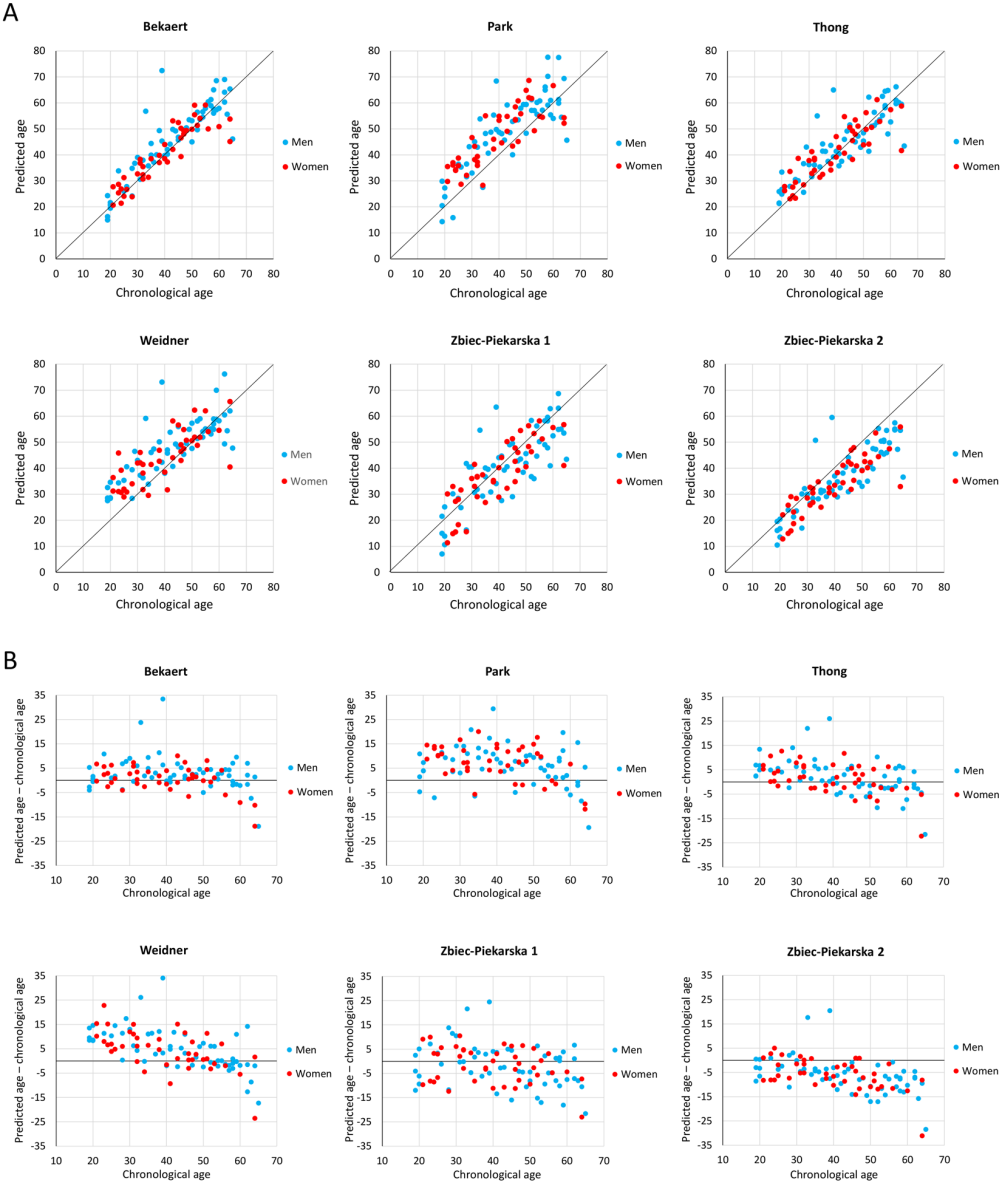
Comparison of the predicted ages obtained with the six age-prediction models. (**A**) Scatterplot of predicted age and chronological age obtained with the six age-prediction models. (**B**) Differences between chronological age and predicted age plotted against chronological age.

The predicted age obtained with the six age-prediction models was plotted against the chronological age (Fig. 2A). The first observation for all age prediction models was that there was no visible and statistically significant difference between men and women for their predicted age, indicating that the six models are not in fact influenced by gender (Fig. 2A, Supplementary Table 4), as had been assumed in the original studies where the models were developed. Correlation analysis indicated a strong correlation (0.783≤ r ≤0.883) between predicted and chronological age for the six models, which explained 61.3% to 77.8% of the age variation (Table 3). The Weidner model showed the lowest correlation in all individuals (r = 0.783) and in women (r = 0.755), and the second lowest correlation in men (r = 0.792). The Bekaert model, in contrast, presented the highest correlation in men (r = 0.883), in women (r = 0.888) and in all individuals (r = 0.883) (Table 3).

**Table 3:** Correlation between chronological age and predicted age obtained with the six age prediction models.

Age prediction model	All		Men		Women	
	r	R²	r	R²	r	R²
Bekaert	0.883	0.780	0.883	0.779	0.888	0.789
Park	0.831	0.690	0.842	0.709	0.810	0.656
Thong	0.853	0.727	0.848	0.719	0.854	0.729
Weidner	0.783	0.613	0.792	0.627	0.755	0.570
Zbiec.Piekarska 1	0.804	0.646	0.790	0.625	0.820	0.672
Zbiec.Piekarska 2	0.856	0.732	0.852	0.725	0.857	0.734

When the differences between chronological and predicted age were plotted for the six models, we observed that some models presented overestimations or underestimations of different magnitudes for predicted age compared to chronological age, and these over/under-estimations also seemed to be influenced by chronological age (Fig. 2B). Therefore we divided our cohort into three groups composed of young adults (Group I, aged 19–34 years, n = 34), middle-aged adults (Group II, aged 35–49 years, n = 33) and older adults (Group III, aged 50–65 years, n = 33), and we measured the mean and median differences between the predicted and the chronological age of the different groups (Supplementary Table 3). Contrary to gender, statistically significant differences were observed for all models between the three age groups indicating that the models have different capacity of age prediction depending on the age range of the samples (Supplementary Fig. 4). The models of Bekaert, Thong and Zbiec-Pierkarska 1 presented very slight over- and under-estimations of the predicted age compared to the chronological age with mean and median age differences of about 2.5 years or less when all individuals were considered (Fig. 2B, Supplementary Table 3). Moreover, these models all tended to slightly overestimate the age of younger individuals and to underestimate the age of older individuals (Fig. 2B, Supplementary Table 3). The Park and Weidner models presented overall overestimations of the predicted age (mean and median over-estimation of 4.74–7.41 years), which were stronger in younger individuals (mean and median over-estimation of 7.50–9.61 years, Fig. 2B, Supplementary Table 3). Finally, the Zbiec-Pierkarska 2 model tended to underestimate the predicted age (mean and median underestimation of 5.99 and 6.41 years respectively) more often in older individuals (mean and median under-estimation of around 10 years) than in younger individuals (mean and median underestimation of around 2 years, Fig. 2B, Supplementary Table 3).

The performance and accuracy of the six age prediction models were evaluated by calculating the mean absolute deviation (MAD), the standard error of estimate (SEE) and the percentage of correct predictions (PCP), considering a difference of 5, 7.5 and 10 years between the predicted and chronological ages for all individuals, as well as for men and for women, and for the three groups based on their chronological age (Table 4). When all individuals were considered, the models of Bekaert and Thong presented the best performance (MAD of 4.5 and 5.2 and SEE of 6.8 and 7.1), while the models of Zbiec-Piekarska 1 & 2 and Weidner presented a lower performance (MAD of 6.8–7.2 and SEE of 8.6–9.6) and the model of Park presented the lowest performance of all (MAD of 8.7 and SEE of 10.2); the same tendencies were observed when men and women were analyzed in two distinct groups (Table 4). Notably, the model of Bekaert, together with the models of Zbiec-Piekarska 2, Thong and Weidner showed the best performance for young adults (MAD of 4.2 and SEE of 5.8–6.3), middle-age adults (MAD of 4.5–4.7 and SEE of 6.8–7.6) and older adults (MAD of 4.7–4.9 and SEE of 6.8–7.7) respectively (Table 4). The poorest performance was observed in the groups of the young and middle-age adults with the Park and Weiner models (MAD of 8.9–9.9 and SEE of 10.3–11.8); while in older adults the poorest performance was observed with the Zbiec-Piekarska 2 model (MAD of 10.5 and SEE of 12.6, Table 4).

**Table 4:** Evaluation of the accuracy of the six age prediction models.

Model	Characteristic		All	Men	Women	Group I	Group II	Group III
Bekaert	MAD		4.5	4.8	4.0	4.2	4.5	4.7
	SEE		6.8	7.6	5.6	6.3	7.6	6.8
	Correct prediction (%)	≤5 years	69	69	69	65	73	70
		≤7.5 years	86	84	88	88	88	82
		≤10 years	92	91	93	94	91	91
Park	MAD		8.7	8.3	9.1	8.9	9.7	7.4
	SEE		10.3	10.2	10.6	10.3	11.5	9.5
	Correct prediction (%)	≤5 years	29	33	24	26	24	36
		≤7.5 years	47	52	40	47	39	55
		≤10 years	63	69	55	56	58	76
Thong	MAD		5.2	5.5	4.9	5.7	4.7	5.3
	SEE		7.2	7.7	6.6	7.6	6.8	7.6
	Correct prediction (%)	≤5 years	55	55	55	53	55	58
		≤7.5 years	82	83	81	76	88	82
		≤10 years	87	86	88	82	94	85
Weidner	MAD		7.2	7.3	7.0	9.9	6.6	4.9
	SEE		9.6	10.0	9.3	11.8	9.5	7.7
	Correct prediction (%)	≤5 years	45	45	45	21	52	64
		≤7.5 years	59	57	62	35	64	79
		≤10 years	69	66	74	53	73	82
Zbiec-Piekarska 1	MAD		6.8	7.3	6.2	6.9	6.2	7.4
	SEE		8.6	9.4	7.6	8.5	8.1	9.7
	Correct prediction (%)	≤5 years	47	48	45	41	58	42
		≤7.5 years	64	62	67	59	73	61
		≤10 years	78	72	86	76	79	79
Zbiec-Piekarska 2	MAD		7.2	7.7	6.5	4.2	7.0	10.5
	SEE		9.2	9.7	8.7	5.8	8.6	12.6
	Correct prediction (%)	≤5 years	40	41	38	62	42	15
		≤7.5 years	59	57	62	82	61	33
		≤10 years	73	71	76	94	76	48

Intergroup comparisons were assessed by Student’s T-tests (see Supplementary Table 4).

When a threshold of 5 years was chosen, and regardless of how the individuals were grouped, the age prediction accuracy was best in the Bekaert model (65–73% of correct predictions), while higher thresholds identified both the Bekaert and Thong models as giving the best age prediction accuracies (76–94% of correct predictions, followed by Zbiec-Piekarska 1 model (59−86% of correct predictions, Table 4). The age prediction accuracies of the Weidner and Zbiec-Piekarska 2 models were highest in the young (62–94% of correct predictions) and older (64–82% of correct predictions) adults regardless of the threshold applied, and were lowest in the older (21–53% of correct predictions) and young (15–48% of correct predictions) adults (Table 4). Finally, the Park model presented an overall low age prediction accuracy for all groups (24–36% of correct predictions with a threshold of 5 years), and this was less pronounced in the group of men and in the older individuals (Table 4).

In order to evaluate the impact of a second measure of DNA methylation on the age prediction performance, we performed a duplicate PCR and pyrosequencing experiment for *ELOVL2* on all samples and compared the age predictions obtained with each replicate and with the mean of duplicates (Supplementary Fig. 6). While the age predictions calculated from each replicate dataset showed similar performances (MAD = 5.8–6.8 and SEE = 7.8–8.6), an improvement was observed when the predicted age was calculated with the mean of duplicates (MAD = 5.2 and SEE = 6.8) (Supplementary Fig. 6).

## Discussion

In the present study, we evaluated six blood-based age prediction models using DNA methylation analysis by pyrosequencing on 100 blood samples from French individuals, categorized by gender and age. We started by implementing the eleven published pyrosequencing assays, analyzing 52 CpG sites using DNA standards with known DNA methylation values. This revealed the presence of a strong bias in favor of the unmethylated allele for *PDE4C*, while for the other assays no or only slight PCR biases were observed (Supplementary Figs 2 and 3). However, the models using *PDE4C* i.e. the models of Bekaert and Weidner, did not show strong age prediction biases in our study suggesting that the DNA methylation bias of *PDE4C* assay could already be present in the original studies. Moreover, these results could not be compared to the original studies as none of the original studies presented pyrosequencing results based on DNA standards. However they could be useful for the future implementation and calibration of these pyrosequencing assays in other laboratories. Compared to the original studies, we used buffy coats for DNA extraction, 1 µg of DNA for bisulfite treatment and 50 cycles for PCR amplification instead of whole blood, 200 ng to 2 µg of DNA and 40–45 cycles respectively^21,22,25–28^. Although these changes should have minor effects on the quantification of DNA methylation and the predicted age, some of them (the use of buffy coats and 1 µg of DNA for bisulfite treatment) could hardly be applied to forensics where only bloodstains are sometimes available.

The correlation analysis between the DNA methylation of each CpG and chronological age also revealed that all CpGs included in the six age-prediction models presented a strong correlation with chronological age, with the notable exception of *ITGA2B* (r = −0.341, Table 2), which is included in the Weidner model^22^. The absence of a correlation between *ITGA2B* methylation and chronological age has already been observed by Bekaert *et al*., who excluded this gene from their model^25^.

The correlation analysis of the six selected models revealed a strong correlation between the predicted and chronological age, with the Bekaert model (r = 0.883) presenting the best observed correlation, followed by the models of Zbiec-Piekarska 2 (r = 0.856), Thong (r = 0.853), Park (r = 0.831), Zbiec-Piekarska 1 (r = 0.804) and Weidner (r = 0.783, Table 3). However, none of the calculated r coefficients exceeded 0.9 in our study, whereas in the original studies establishing these models, the r coefficients ranged from 0.927 to 0.977 in the training sets as well as in the validation sets (Supplementary Table 4)^21,25–28^. The lower r coefficients obtained in our study could principally be explained by the smaller age range of our cohort (19–65 years) compared to the original studies (0–91 years, Supplementary Table 4)^21,22,25–28^. This phenomenon has already been described in a previous study where the use of restricted ranges of data resulted in a decrease of the r coefficient^32^.

Of the six models, the Bekaert model presented the best overall performance and accuracy for age prediction (MAD of 4.5 and SEE of 6.8, Table 4) in our cohort of 100 French blood samples, closely followed by the Thong model (MAD of 5.2 and SEE of 7.2). Thus both models presented overall performances close to the original studies (MAD of 3.75 for the Bekaert model and 3.3–5 for the Thong model, Supplementary Table 4)^25,26^, suggesting small inter-laboratory variations.

The Zbiec-Piekarska 1 model is the only age prediction model based solely on 2 CpGs located at a single locus and requiring only one PCR and pyrosequencing assay, thus rendering this model particularly useful for rapid age estimation when only low quantities of DNA are available such as in forensic applications. This model presented a good overall performance (MAD of 6.8 and SEE of 8.6, Supplementary Table 4), although slightly below the performances obtained in the original study (MAD of 5.03 and 5.75 in both the training and validation sets respectively, Supplementary Table 4)^27^. However, we showed for the first time that the use of the mean of two replicates for the quantification of DNA methylation rather than a single replicate improved the performance of age prediction of Zbiec-Piekarska 1 model. This suggests that the use of duplicate measures of DNA methylation could potentially be a simple way to increase the performance of age prediction for every model. The Weidner model, which was the first blood-based age-prediction model to be developed using pyrosequencing, presented one of the lowest performances for age prediction (MAD of 7.2 and SEE of 9.6) with an overestimation of the predicted age in younger adults (Table 4). This model gave lower performances to those of the original study (MAD of 4.49–5.43 and RMSE of 5.6–7.2, Supplementary Table 4), where slight over-estimations of the predicted age in younger individuals were also visible^22^.

The overall accuracy observed for the Zbiec-Piekarska 2 and Park models was among the lowest out of the six models tested (MAD of 7.2–8.7 and SEE of 9.2–10.3, Table 4), which contrasted with the better values of the performance indicators for these models in the original studies (MAD of 3.1–3.9 and SEE of 4.5–6.9, Supplementary Table 4)^21,28^. Two independent studies also evaluated the Zbiec-Piekarska 2 model and found a higher MAD (4.18 and 4.8) compared to the original study^26,29^, suggesting that inter-laboratory variability can influence the performance of a model. However, in our study, the models of Zbiec-Piekarska 2 and Park tended to systematically under- and over-estimate the predicted age compared to the chronological age (Fig. 2, Supplementary Table 3), a finding not observed in the original studies^21,28^. Analysis of the raw pyrosequencing data from the original studies of the Park^21^ and Zbiec-Piekarska 2 models^28^ indicated that our pyrosequencing data for *ELOVL2* CpG1 and *C1orf132* CpG1 presented a higher level of DNA methylation for all age groups, which may have potentially induced a systematic over- and under-estimation of the predicted age respectively (not shown). Thus, to avoid this potential technical bias, we recommend that DNA methylation data on standards with known DNA methylation levels should be provided in the original studies in which the age-prediction models are developed, and that a systematic calibration of each pyrosequencing assay based on the evaluation of the above-mentioned standards should be performed in different laboratories during implementation of the pyrosequencing assays and the age prediction models, as slight variations of the experimental conditions (quantity of input DNA, annealing temperature, pyrosequencing instruments…) from one laboratory to another can modify the quantification of DNA methylation^27,30^.

Finally, we also observed in our study that gender had little or no effect on age prediction accuracy, which is consistent with reports in previous studies^21,25,28^. However, while an increase in the chronological age of the individuals has been shown to negatively influence age prediction accuracy in most studies to date^21,25,26,28^, this observation was not clearly visible in our own study, a fact that could probably be explained by the narrow age range of our cohort. Due to the limited number of individuals included in our study, our conclusions on the six blood-based age prediction models should be further confirmed and strengthened in other validation studies using larger cohorts.

## Conclusion

This study constitutes the first independent evaluation and validation based on blood samples of 100 individuals from a French cohort, tested with six previously developed blood-based age prediction models using DNA methylation analysis by pyrosequencing, with the aim of performing a direct intercomparison of the six models in addition to comparing the results to the original studies in which the models were developed. The study notably revealed various differences in performance for age prediction in the six models, with the models of Bekaert and Thong presenting the best age prediction accuracy. The model of Zbiec-Piekarska 1 was the easiest to implement, being based on 2 CpGs included in a single pyrosequencing assay, and presented a good performance for age prediction which could prove particularly useful for forensic applications where the amount of available DNA is limited. In our hands, the models of Park and Zbiec-Piekarska 2 presented slight over- and under-estimation biases in predicted age compared to chronological age, probably caused by inter-laboratory variations during implementation of the pyrosequencing assays. Thus age-prediction models developed in the future should include DNA methylation data on standards with known DNA methylation values for every pyrosequencing assay to facilitate the implementation of these models in other laboratories. Moreover, a systematic evaluation of the different age prediction models on a same population should be performed in order to compare their performance and identify the model with the best age prediction accuracy.

## Supplementary information

Supplementary Information

Supplementary Dataset 1
