# Supplementary material for: Evaluation of six blood-based age prediction models using DNA methylation analysis by pyrosequencing
Source: Sci Rep. 2019 Jun 20;9:8862. doi: 10.1038/s41598-019-45197-w (PMC6586942; doi:10.1038/s41598-019-45197-w)
Supplement: Supplementary file 1 — Supplementary Information [file 41598_2019_45197_MOESM1_ESM.pdf]

Supplementary information for:

Evaluation of six blood-based age prediction models using DNA methylation analysis by pyrosequencing

Antoine Daunay[1], Laura G. Baudrin[1,2], Jean-François Deleuze[1,3] & Alexandre How-Kit[1‡]

[1] Laboratory for Genomics, Foundation Jean Dausset – CEPH, Paris, France

[2] Laboratory of Excellence GenMed, Paris, France

[3] Centre National de Recherche en Génomique Humaine, CEA-Institut François Jacob, Evry, France

[‡] ***Correspondence to:***

Alexandre How-Kit, Ph.D., Laboratory for Genomics, Foundation Jean Dausset - CEPH, Paris, F-75010, France, Tel.: +33-(0)1- 53725146, email: alexandre.how-kit@fjd-ceph.org

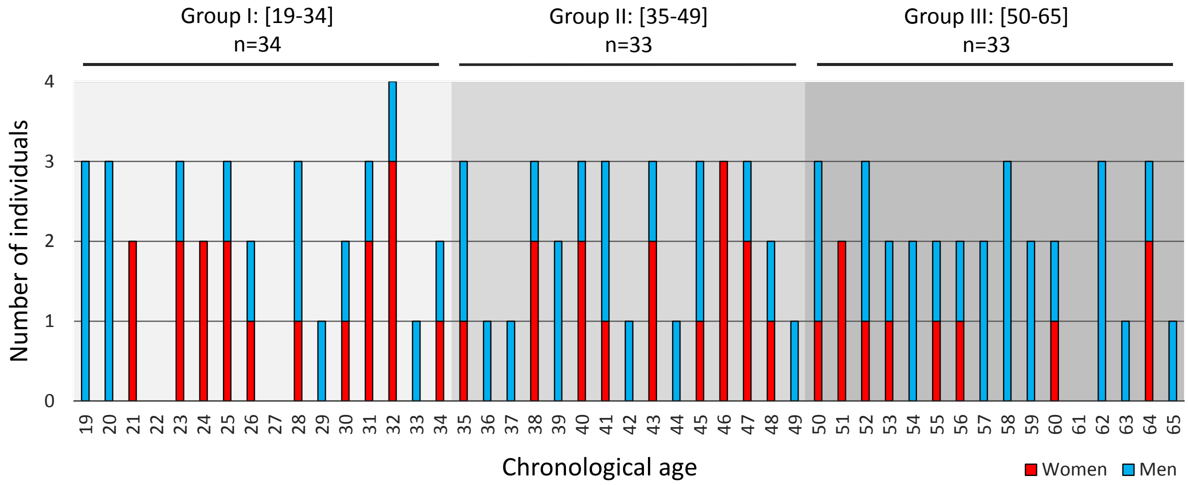

Supplementary Figure 1: Age and sex distribution of the cohort of 100 healthy blood donors

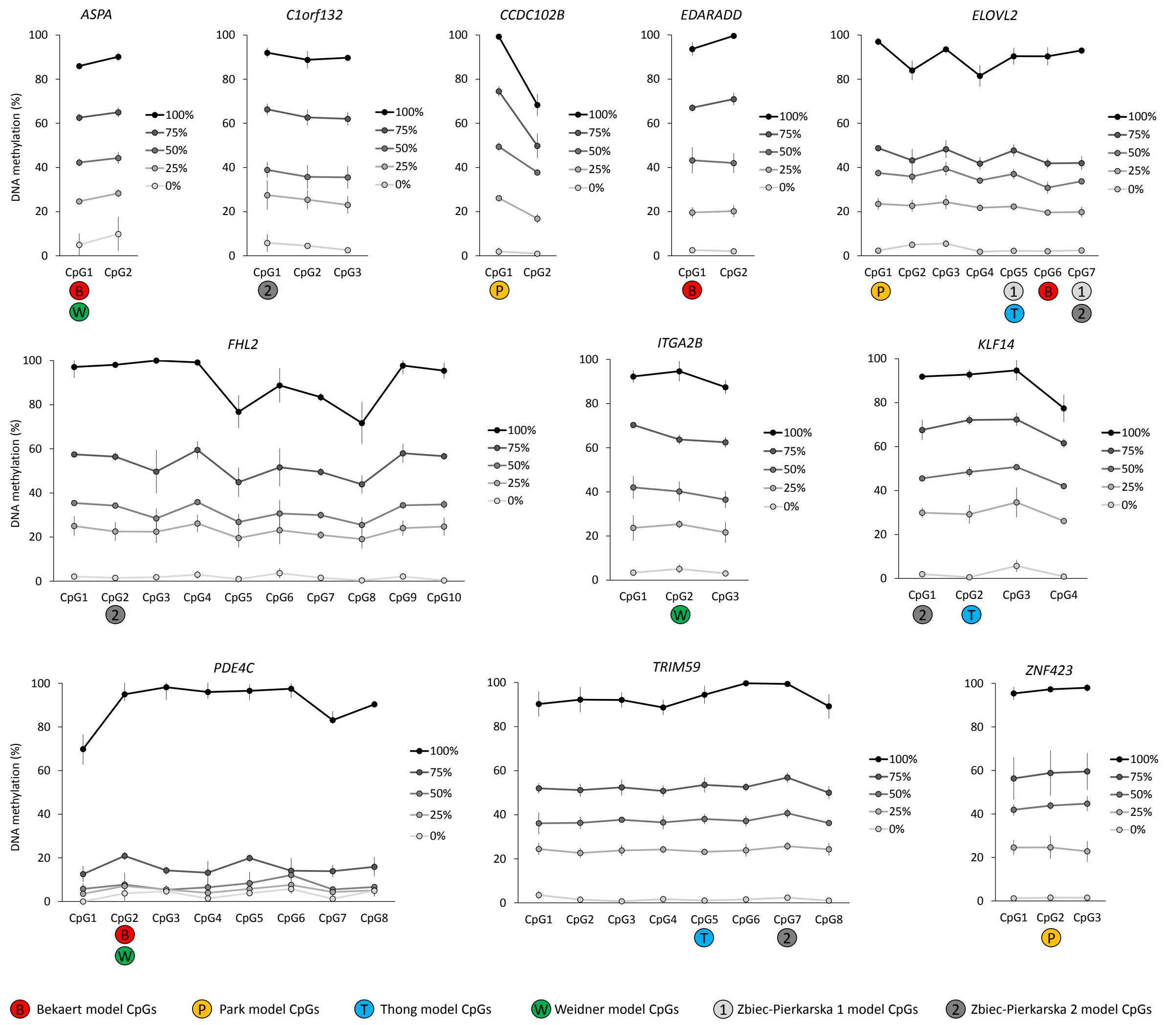

Supplementary Figure 2: DNA methylation patterns of all analyzed CpGs for all genes using 0%, 25%, 50%, 75% and 100% DNA methylation standards (the 0% and 100% DNA methylation standards were purchased from Qiagen and mixed in 3:1, 1:1 and 1:3 equimolar ratios to obtain 25%, 50% and 75% DNA methylation standards). The CpGs included in the age prediction models are indicated below the relevant CpGs. Experiments were performed in triplicate and vertical bars in the line graphs represent standard deviation.

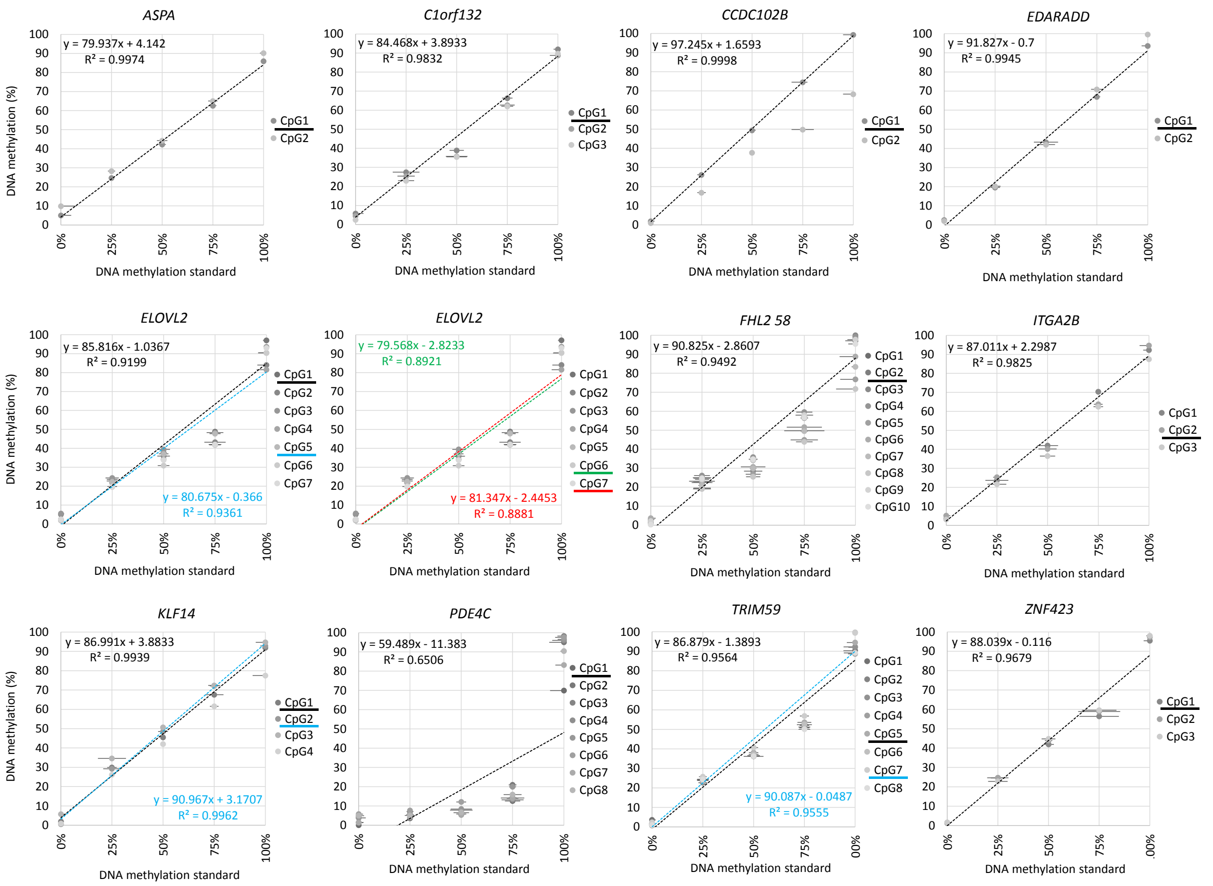

Supplementary Figure 3: Dot plots of the expected and obtained DNA methylation values per gene for all analyzed CpGs. using 0%, 25%, 50%, 75% and 100% DNA methylation standards (the 0% and 100% DNA methylation standards were purchased from Qiagen and mixed in 3:1, 1:1 and 1:3 equimolar ratios to obtain 25%, 50% and 75% DNA methylation standards). Experiments were performed in triplicate and horizontal bars in the plots represent standard deviation. The linear regression equations and the coefficients of determination R^2 are indicated for the CpGs (underlined) included in the age prediction models.

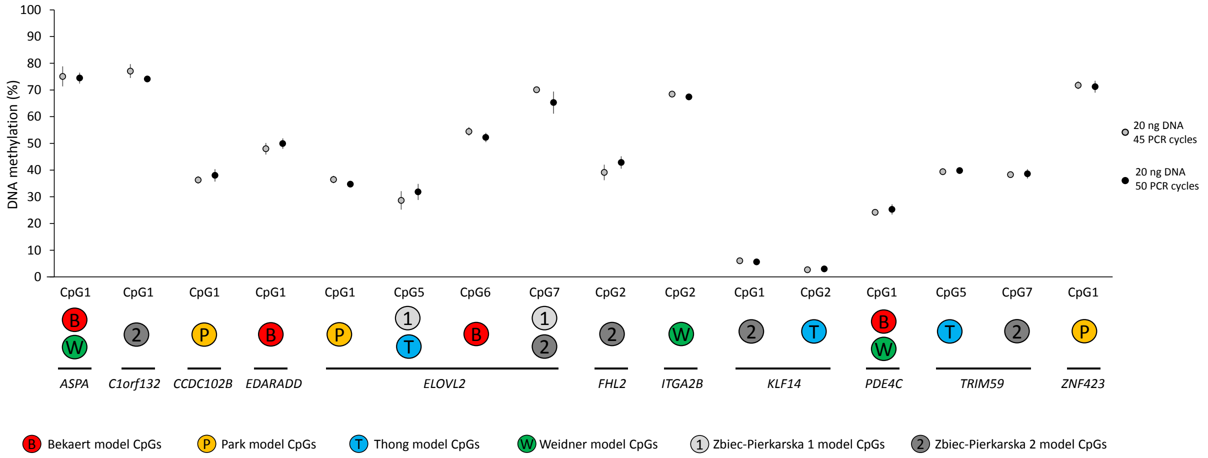

Supplementary Figure 4: Effect of the quantity of DNA template and of the number of PCR cycles on DNA methylation using a bisulfite-treated commercial whole blood DNA sample (Promega). Experiments were performed in triplicate and vertical bars in the graph represent standard deviation. Differences between groups were assessed using Student's T-test and were not significant (*p*<0.05 threshold).

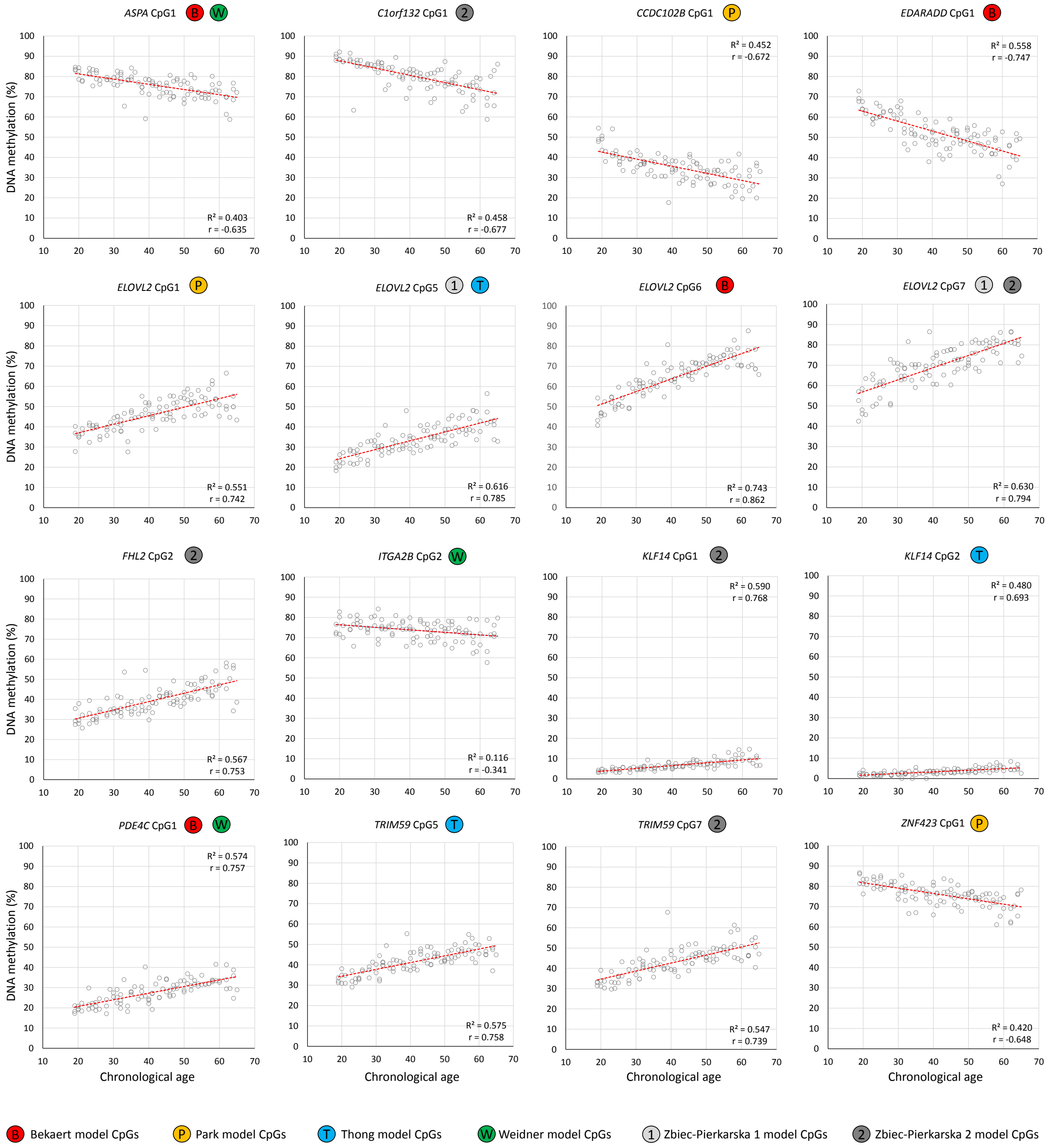

Supplementary Figure 5: Variation of the DNA methylation of the CpG markers included in the six blood-based age prediction models according to the chronological age of the 100 French individuals.

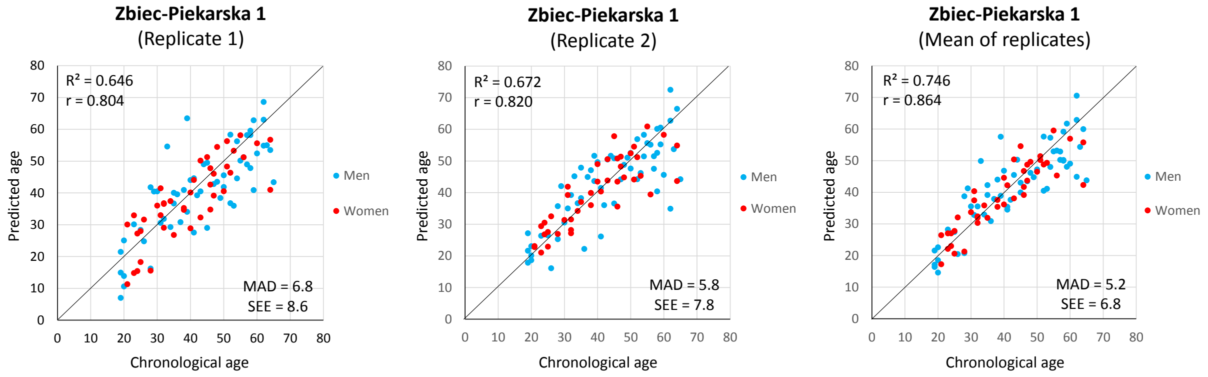

Supplementary Figure 6: Effect of pyrosequencing experiments performed in duplicates on age prediction accuracy of the model of Zbiec-Piekarska 1. Two independent PCR and pyrosequencing experiments were performed on all samples to obtain both measures of DNA methylation. R^2, coefficient of determination; r, Pearson correlation coefficient; MAD, mean absolute deviation; SEE, standard error of the estimate.

Supplementary Table 1: List of PCR primers, pyrosequencing primers and sequences for analysis

Gene	Primer description	Primer sequence	Ta[#]	Sequence to analyze[§]	Ref.
ASPA	PCR_Forward	Biotin-ATTATTTGGTGAAATGATT	52°C	ACR$_{1}$CCATTCTCTAACCAATACTTAACCAAAAATACTCCR$_{2}$ATTAACT	1
	PCR_Reverse	CAACCCTATTCTCTAAATCTC			
	Pyrosequencing	CCCTATTCTCTAAATCTCA	-		
C1orf132	PCR_Forward	Biotin-GTAAATATATAAGTGGGGGAAGAAGGG	55°C	AAATCTACR$_{1}$CAAACR$_{2}$ACR$_{3}$ATAAATAATCC	2
	PCR_Reverse	TTAATAAAACCAAATTCTAAAACATTC			
	Pyrosequencing	CACCTTACCACCAAACCAAAATTT	-		
CCDC102B	PCR_Forward	GGGAGGGGAATGTTTGTATTTAT	60°C	TTTYG$_{1}$TATTTTTTTTTTGGTTGTTATTTTTGYG$_{2}$GGGATTGTTT	3
	PCR_Reverse	Biotin-TCCTTTCTTTACTACATTCCA			
	Pyrosequencing	AGGGGAATGTTTGTATTTA	-		
EDARADD	PCR_Forward	TTGGTGATTAGGAGTTTTAGTGTTTT	56°C	YG$_{1}$AGAAGATGTTYG$_{2}$TTGG	4
	PCR_Reverse	Biotin-CCACCTACAAATTCCCCAAA			
	Pyrosequencing	GGAGTTTGTTATGGAAGAAGTAATAGATTG	-		
ELOVL2	PCR_Forward	Biotin-AGGGGAGTAGGGTAAGTGAGG	60°C	CCR$_{1}$TGAAACR$_{2}$TTGAAGACCR$_{3}$CCR$_{4}$CR$_{5}$CR$_{6}$AAACCR$_{7}$AC	5
	PCR_Reverse	AACAAAACCATTTCCCCCTAATAT			
	Pyrosequencing	ACAACCAATAAATATTCCTAAAACT	-		
FHL2	PCR_Forward	TGTTTTTAGGGTTTTGGGAGTATAG	58°C	AGTTATYG$_{1}$GGAGYG$_{2}$TYG$_{3}$TTTTYG$_{4}$GYG$_{5}$TGGGTTTTYG$_{6}$GGYG$_{7}$YG$_{8}$AGTTTYG$_{9}$GAYG$_{10}$AGGTTT	2
	PCR_Reverse	Biotin-ACACCTCCTAAAACTTCTCCAATCTCC			
	Pyrosequencing	ATAGTTTTAGAAATTATTTTGTTT	-		
ITGA2B	PCR_Forward	Biotin-TAATTTTTTTTGGGTGATG	53°C	ACR$_{1}$TATATTAACCCACR$_{2}$CR$_{3}$ACCAACC	1
	PCR_Reverse	ACCAAAAATAAACAATATACTCAAT			
	Pyrosequencing	CAATATACTCAATACTATACCT	-		
KLF14	PCR_Forward	GGTTTTTAGGTTAAGTTATGTTTAATAGT	58°C	TYG$_{1}$YG$_{2}$TTTTTTTTTTTTGTYG$_{3}$GYG$_{4}$AGTTAGGTAATGG	2
	PCR_Reverse	Biotin-ACTACTACAACCCAAAAATTCC			
	Pyrosequencing	ATAGTTTTAGAAATTATTTTGTTT	-		
PDE4C	PCR_Forward	AGGTTTGTAGTAGGTTGAG	53°C	YG$_{1}$AAGTATTTGTGGYG$_{2}$GTAATTTYG$_{3}$GYG$_{4}$TTTTATTYG$_{5}$TATTTAATAGYG$_{6}$TTTTTATTYG$_{7}$GATTYG$_{8}$GATAAG	1
	PCR_Reverse	Biotin-AACTCAAATCCCTCTC			
	Pyrosequencing	GTTATAGTATGATTAGAGTTT	-		
TRIM59	PCR_Forward	TATAGGTGGTTTGGGGGAGAG	58°C	GGTTTGGYG$_{1}$YG$_{2}$GGAYG$_{3}$AGGYG$_{4}$AAGYG$_{5}$TYG$_{6}$GTGGTYG$_{7}$AYG$_{8}$GTTTTTGAGGAA	2
	PCR_Reverse	Biotin-AAAAAACACTACCCTCCACAACATAAC			
	Pyrosequencing	TTGGGGGAGAGGTTG	-		
ZNF423	PCR_Forward	Biotin-GAGGGAGATAGTAGATGTGTTGTGTA	60°C	CCR$_{1}$CR$_{2}$TCTTCCTACCR$_{3}$AATAACCTATCCCCT	3
	PCR_Reverse	CCCACCTAAACCCTAAATTCCTAAA			
	Pyrosequencing	CAACAACAAAAACTCCTC	-		

[#] Ta, primer annealing temperature.

[§] each CpG is numbered in the same order as in the article

Supplementary Table 2: Description of the genes used and genomic coordinates of the analyzed CpGs

Gene Symbol	Full gene name	CpG	Chromosome location (GRCh38)
ASPA	Aspartoacylase	1	Chr17: 3,476,273
		2	Chr17: 3,476,237
C1orf132	-	1	Chr1: 207,823,681
		2	Chr1: 207,823,675
		3	Chr1: 207,823,672
CCDC102B	Coiled-coil domain containing 102B	1	Chr18: 68,722,183
		2	Chr18: 68,722,210
EDARADD	EDAR associated death domain	1	Chr1: 236,394,382
		2	Chr1: 236,394,370
ELOVL2	ELOVL fatty acid elongase 2	1	Chr6: 11,044,661
		2	Chr6: 11,044,655
		3	Chr6: 11,044,647
		4	Chr6: 11,044,644
		5	Chr6: 11,044,642
		6	Chr6: 11,044,640
		7	Chr6: 11,044,634
FHL2	Four and a half LIM domains 2	1	Chr2: 105,399,282
		2	Chr2: 105,399,288
		3	Chr2: 105,399,291
		4	Chr2: 105,399,297
		5	Chr2: 105,399,300
		6	Chr2: 105,399,310
		7	Chr2: 105,399,314
		8	Chr2: 105,399,316
		9	Chr2: 105,399,323
		10	Chr2: 105,399,327
ITGA2B	Integrin subunit alpha 2b	1	Chr17: 44,390,375
		2	Ch17: 44,390,361
		3	Ch17: 44,390,359
KLF14	Kruppel like factor 14	1	Chr7: 130,734,355
		2	Chr7: 130,734,357
		3	Chr7: 130,734,373
		4	Chr7: 13,0734,375
PDE4C	Phosphodiesterase 4C	1	Ch19: 18,233,106
		2	Ch19: 18,233,092
		3	Ch19: 18,233,083
		4	Ch19: 18,233,080
		5	Ch19: 18,233,071
		6	Ch19: 18,233,059
		7	Ch19: 18,233,049
		8	Ch19: 18,233,043
TRIM59	Tripartite motif containing 59	1	Chr3: 160,450,172
		2	Chr3: 160,450,174
		3	Chr3: 160,450,179
		4	Chr3: 160,450,184
		5	Chr3: 160,450,189
		6	Chr3: 160,450,192
		7	Chr3: 160,450,199
		8	Chr3: 160,450,202
ZNF423	Zinc finger protein 423	1	Ch16: 49,491,896
		2	Ch16: 49,491,894
		3	Ch16: 49,491,883

Supplementary Table 3: Mean and median values of predicted age minus chronological age in the six age-prediction models.

Individuals	Bekaert		Park		Thong		Weiner		Zbiec-Piekarska 1		Zbiec-Piekarska 2	
	**Mean**	**Median**	**Mean**	**Median**	**Mean**	**Median**	**Mean**	**Median**	**Mean**	**Median**	**Mean**	**Median**
All	2.03	1.84	6.65	7.41	1.67	1.41	4.74	4.80	-1.86	-2.59	-5.99	-6.41
Men	2.99	2.05	6.11	6.84	1.88	1.41	5.04	5.03	-2.01	-2.62	-6.14	-6.51
Women	0.69	1.07	7.40	7.96	1.37	1.43	4.32	4.74	-1.65	-1.82	-5.80	-5.95
Group I [19 - 34 years]	2.89	1.82	7.50	8.66	5.34	4.93	9.61	9.48	1.16	2.73	-1.98	-2.12
Group II [35 - 49 years]	3.63	2.34	9.16	8.04	2.01	0.97	5.33	4.66	-1.35	-2.48	-5.65	-6.24
Group III [50 - 65 years]	-0.47	0.74	3.27	4.13	-2.46	-2.37	-0.88	-1.16	-5.47	-4.82	-10.46	-10.27

The values are highlighted in color on a green-yellow-red scale.

Supplementary Table 4: T-test results (*p-values*) of comparisons obtained for the six age-prediction models.

Model	Men vs Women	Group I vs Group II	Group II vs Group III	Group I vs Group III
Bekaert	0.0670	0.6192	**0.0140**	**0.0269**
Park	0.4038	0.3090	**0.0029**	**0.0302**
Thong	0.7112	**0.0224**	**0.0089**	**<0.0001**
Weidner	0.6688	**0.0149**	**0.0014**	**<0.0001**
Zbiec-Piekarska 1	0.8289	0.2095	**0.0357**	**0.0012**
Zbiec-Piekarska 2	0.8038	**0.0118**	**0.0030**	**<0.0001**

For each comparison, a Student's T-test was performed using the values of the predicted age minus chronological age of each group. The *p-values* less or equal to 0.05 are shown in bold.

Supplementary Table 5: Performance of the six age-prediction models in the training and validation sets as given in the original studies in which they were developed.

Models	Training set					Validation set				
	Blood samples	Age range	Correlation coefficient r[a]	MAD	SEE or RMSE	Blood samples	Age range	Correlation coefficient r[a]	MAD	SEE or RMSE
Bekaert[4]	206	0-91	0.97	3.75	-	-	-	-	-	-
Park[3]	535	11-90	0.956	3.156	6.32	230	11-90	0.954	3.346	6.853
Thong[6]	145	3-80	0.977	3.3	-	116	0-88	-	5	-
Weidner[1]	82	0-75	-	5.43	7.2	69	20-75	-	4.49	5.6
Zbiec-Piekarska 1[5]	303	2-75	0.927	5.03	-	124	2-75	0.931	5.75	-
Zbiec-Piekarska 2[2]	300	2-75	0.971	3.4	4.5	120	2-75	0.972	3.9	-

[a] the coefficient correlation r is sometimes calculated as the square root of the coefficient of determination provided in the original studies.

"-" indicates an absence of data in the original paper; r = Pearson correlation coefficient; MAD = mean absolute deviation; SEE = standard error of the estimate; RMSE = root mean square error.

References

1 Weidner, C. I. *et al.* Aging of blood can be tracked by DNA methylation changes at just three CpG sites. *Genome biology* 15, R24, doi:10.1186/gb-2014-15-2-r24 (2014).

2 Zbiec-Piekarska, R. *et al.* Development of a forensically useful age prediction method based on DNA methylation analysis. *Forensic science international. Genetics* 17, 173-179, doi:10.1016/j.fsigen.2015.05.001 (2015).

3 Park, J. L. *et al.* Identification and evaluation of age-correlated DNA methylation markers for forensic use. *Forensic science international. Genetics* 23, 64-70, doi:10.1016/j.fsigen.2016.03.005 (2016).

4 Bekaert, B., Kamalandua, A., Zapico, S. C., Van de Voorde, W. & Decorte, R. Improved age determination of blood and teeth samples using a selected set of DNA methylation markers. *Epigenetics* 10, 922-930, doi:10.1080/15592294.2015.1080413 (2015).

5 Zbiec-Piekarska, R. *et al.* Examination of DNA methylation status of the ELOVL2 marker may be useful for human age prediction in forensic science. *Forensic science international. Genetics* 14, 161-167, doi:10.1016/j.fsigen.2014.10.002 (2015).

6 Thong, Z., Liang Shun Chan, X., Ying Ying Tan, J., Shuzhen Loo, E. & Kiu Choong Syn, C. Evaluation of DNA methylation-based age prediction on blood. *Forensic Science International: Genetics Supplement Series* 6, e249–e251 doi:https://doi.org/10.1016/j.fsigss.2017.09.095 (2017).
